# Neurodevelopment Genes Encoding Olduvai Domains Link Myalgic Encephalomyelitis to Neuropsychiatric Disorders

**DOI:** 10.3390/diagnostics15121542

**Published:** 2025-06-17

**Authors:** Mauricio Arcos-Burgos, Mauricio Arcos-Holzinger, Claudio Mastronardi, Mario A. Isaza-Ruget, Jorge I. Vélez, Donald P. Lewis, Hardip Patel, Brett A. Lidbury

**Affiliations:** 1 Research Group on Psychiatric Disorders (GIPSI), Department of Psychiatry, Institute of Medical Research, School of Medicine, University of Antioquia, Medellín 050010, Colombia; 2 INPAC Research Group, Fundación Universitaria Sanitas, Bogotá 110131, Colombia; 3 Department of Industrial Engineering, Universidad del Norte, Barranquilla 081007, Colombia; 4 CFS Discovery, Donvale Specialist Medical Centre, Donvale, VIC 3111, Australia; 5 John Curtin School Medical Research, ANU College of Health and Medicine, The Australian National University, Canberra, ACT 2601, Australia; 6 National Centre for Epidemiology and Population Health (NCEPH), ANU College of Health and Medicine, The Australian National University, Canberra, ACT 2601, Australia

**Keywords:** Myalgic Encephalomyelitis (ME), Olduvai (DUF1220), exome, neurodevelopment

## Abstract

**Background/Objectives:** The aetiology of Myalgic Encephalomyelitis/Chronic Fatigue Syndrome (ME/CFS), a chronic and severe debilitating disease with a complex phenotype, remains elusive. Associations with infectious diseases and autoimmune and neuropsychiatric disorders have been observed, without the identification of mechanisms. Previous studies suggest that genetic predisposition plays a role, but results are difficult to replicate, with Genome-Wide Association Studies of ME/CFS being challenging due to the relative rareness and heterogeneity of the disorder. **Methods:** We studied a well-defined Australian patient cohort diagnosed via the International Consensus Criteria, recruited by a specialist ME/CFS clinic. The whole-exome sequences of 77 patients were contrasted against genome variation in the 1000 Genome Project’s genome-matched population. **Results:** Significant associations with ME/CFS were harboured in genes that belong to the Neuroblastoma Breakpoint Family encoding Olduvai (DUF1220) domains, namely *NBPF1* (rs3897177, *p*-value = 3.15 × 10^−8^)*, NBPF10* (rs1553120233, *p*-value = 9.262 × 10^−13^), and *NBPF16* (rs200632836, *p*-value = 1.04 × 10^−6^). Other significantly associated variants were detected in the *ATR*, *RSPH10B*, *ADGRE5-CD97*, and *NTRK2* genes, among others. Replication of these results was attempted via a GWAS on raw data from a US cohort, which confirmed shared significant associations with variation identified in the *PTPRD*, *CSMD3*, *RAPGEF5*, *DCC*, *ALDH18A1*, *GALNT16*, *UNC79*, and *NCOA3* genes. **Conclusions:** These genes are involved in cortical neurogenesis, brain evolution, and neuroblastoma, and have been implicated by several studies in schizophrenia and autism. The sharing of these associations by the two cohorts supports their validity and grants the necessity of future studies to evaluate the implications for ME/CFS aetiology.

## 1. Introduction

Myalgic Encephalomyelitis/Chronic Fatigue Syndrome (ME/CFS or ME) is a complex, multi-system, and chronic debilitating disease characterised by persistent idiopathic fatigue and post-exertional malaise (PEM), which can also involve combinations of muscle pain, immune dysfunction, gut complications, and disorders of sleep and neurocognition [1]. Estimates of its prevalence range between 836,000 and 2.5 million patients (0.2–0.8%) in the United States, with a 0.2% prevalence found in a UK study, and with investigations underway to accurately estimate its European prevalence [2,3,4]. The determination of ME prevalence is hindered by the absence of agreement on diagnostic consensus criteria [5,6], and the lack of a biomarker [7]. Previous studies have identified genetic factors associated with ME/CFS, with links, for example, to infection, autoimmunity, and neuroendocrine function [8,9,10]. Like other aspects of ME/CFS research, though, consensus on genetic results have been difficult to obtain [11,12,13,14,15].

In spite of these challenges, genetic epidemiological studies, including twin and family investigations, have shown the clustering of fatigue and neurological symptoms in mothers that are similar to those exhibited by their ME/CFS affected children [16]. Also, higher concordance rates of ME/CFS were identified in monozygotic twins when compared to dizygotic twins, without pronounced gender differences, in Swedish national twin samples [17]. The estimated heritability ranges from 19% (95% confidence interval = 0–56) to 51% (95% confidence interval = 7–96) [17,18,19], indicating that familial aggregation underpins ME/CFS.

Here we present the results of whole-exome capture and next-generation sequencing of a patient cohort recruited from the greater Melbourne region of south-eastern Australia. Issues with consistency and study replication were addressed through two features of the clinical and research team: (1) All research participants were assessed, diagnosed, and supported by clinicians with 15 years of dedicated and exclusive experience with ME/CFS patients, and who only used the Canadian Consensus Criteria–International Consensus Criteria (ICC) to diagnose and monitor patients [5,20]. An author on this study (D.P.L.) was a member of the panel that published the ICC. (2) The team’s genomics expert (M.A.-B.) specialises in the genetics of complex diseases (e.g., Alzheimer’s Disease) and has led the development of methods that allow the performance of GWAS on small patient sample sizes (pooling/bootstrap methods) [21]. To increase the rigour of our genetic investigation, whole-exome significant associations between genome variants and ME/CFS susceptibility were configured to interactive oligogenic and/or multifactorial models of transmission. These Australian observations were subsequently validated by previous results reported for a Nevada ME/CFS cohort in the United States [22], with identical pathways detected.

Prominent among these pathways were Neuroblastoma Breakpoint Family (*NBPF*) genes encoding Olduvai (DUF1220) domains, located in genes associated with autism [23] and other neurological conditions. The Olduvai domain holds deeper significance through associations with the evolution of higher brain functions in humans, a feature not shared with other primates. The Olduvai name was proposed in recognition of the region in Africa where much seminal work was performed by scientists on early human evolution [24]. It can be inferred from the connection of ME/CFS to the Olduvai domain, joining autism, schizophrenia, and other complex disorders [24,25], that the advanced evolution of human brain function also involved predisposition to unique human diseases.

## 2. Materials and Methods

Participant Recruitment and Ethics Approval: Research participants were recruited via the CFS Discovery Clinic (Donvale, VIC 3111, Australia) via direct invitation to existing patients. Only participants with a previous ME/CFS diagnosis were recruited.

Human ethics approval was granted by the ANU Human Research Ethics Committee (Approval No. 2015/193), with approved consent forms and participant information provided to each potential research participant. Inclusion in the study was allowed after signed consent was received by the researchers. Specific participant identifiers were not supplied to the researchers and were only known to the clinicians and clinic staff. Each research participant was given an identification code by the clinic, with age (at time of the appointment) and sex also provided.

Each consenting participant was examined according to the *International Consensus Criteria* for ME [5] and included in the ME/CFS cohort if the criteria for inclusion were fulfilled. As part of this examination, qualitative symptom inventories and questionnaires were conducted for each participant, namely the Epworth Sleep Scale [26], the DASS-42 [27], and the Multidimensional Fatigue Inventory (MFI) [28]. Other clinical procedures, tests, cohort descriptions, and results have been reported previously, including comparisons with a healthy control group [29] and the calculation of symptom severity via Weighted Standing Time (WST) [30]. For these exome analyses, only the ME/CFS cohort was investigated, with 77 of the 80 participants initially recruited providing consent for this study, as well as meeting all inclusion criteria and availability requirements.

Whole-Exome Capture, Sequencing, Bioinformatics, and Genetic Analysis:

DNA extraction: Five millilitres (5 mL) of venous blood was collected from the forearm vein of consenting study participants (anti-coagulant tetra-sodium EDTA), as per standard phlebotomy practice for pathology samples. Samples were briefly stored at 4 °C before genomic DNA extraction using the QIAGEN DNeasy Blood & Tissue Kit (Cat No.: 69504, QIAGEN Pty Ltd.—Clayton, VIC, Australia), with extracted DNA stored at −80 °C until analysis. Three methods were applied for DNA quantification and qualification: (1) DNA purity was checked using a Nanodrop (OD260/280 ratio) (ThermoFisher Scientific, Melbourne, VIC., Australia); (2) DNA degradation and contamination were monitored on 1% agarose gels; (3) DNA concentration was measured using Qubit (ThermoFisher Scientific, Melbourne, VIC., Australia). DNA samples with OD260/280 ratios between 1.8~2.0 and concentrations above 1.0 ug were used to prepare the sequencing libraries.

Library preparation for sequencing: Agilent liquid-phase hybridization was applied to efficiently enrich the exons, which were sequenced on an Illumina platform (Illumina, Inc., San Diego, CA, USA). The sequencing libraries and capture used Agilent SureSelect Human All ExonV5/V6 (Agilent Technologies, St. Clara, CA, USA) with the reagents recommended by the instruction manual and following experimental procedures for optimal results.

Next-generation sequencing: Genomic DNA was randomly fragmented to 180–280 bp with Covaris cracker, and then DNA fragments were end-polished, A-tailed, and ligated with the full-length adapter for Illumina sequencing (Illumina, San Diego, CA, USA). Fragments with specific indexes were hybridised with more than 543,872 biotin-labelled probes after pooling, and then magnetic beads with streptavidin were used to capture 334,378 exons from 20,965 genes. After PCR amplification and quality control, libraries were sequenced.

Bioinformatics analysis: All the sequenced data were quality assessed (base quality distribution, nucleotide distribution, and the presence of adapters, chimaeras and other contaminants) to identify/remove low-quality data/samples from further analysis. All high-quality data was then mapped to the human genome assembly using the *bwa-mem* algorithm [31,32]. The aligned files were processed using the Genome Analysis Tool Kit (GATK) (https://gatk.broadinstitute.org/hc/, accessed on 10 June 2025) for base quality recalibration, indel realignments, and duplicate removal [33,34]. This was followed by SNP and INDEL discovery and genotyping (plus phasing where applicable) according to GATK Best Practices recommendations [35,36]. All variant calls were subject to variant quality score recalibration and filtering to remove low-quality variants.

**Figure 1:**
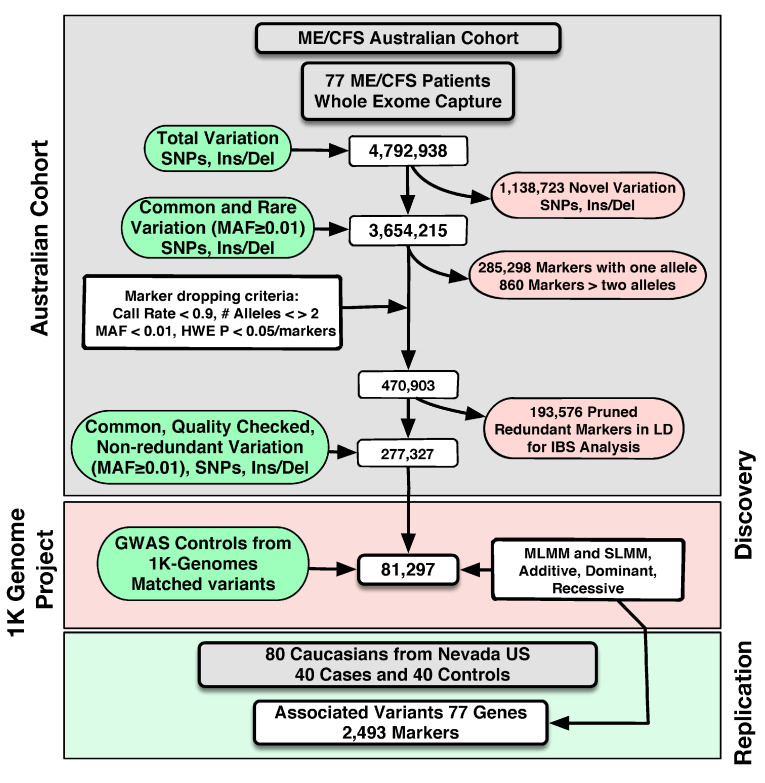
Testing and analysis strategy for ME genomic DNA samples (*n* = 77) subject to whole-exome capture prior to next-generation sequencing, and Genome-Wide Association Studies (GWAS). # = Number (number of Alleles).

Quality control, filtering, and classification of coding variants: Genetic data were imported to Golden Helix^®^’s SVS 8.8.3, and quality control was performed, as recommended by several authors [37,38,39], including specific criteria for ME/CFS [15]. In brief, the steps followed were as follows: (i) fitting to Hardy–Weinberg equilibrium with *p*-values > 0.05/*m* (where *m* is the number of markers included for analysis); (ii) confirming a minimum genotype call rate of 90%; (iii) checking for the presence of two alleles; (iv) evaluating the control of the excess in heterozygosity by the departure from expected *f coefficient* (inbreeding coefficient)*,* i.e., significant deviation toward negative values. Markers not meeting any of these criteria were excluded from analyses. Genotype and allelic frequencies were estimated by maximum likelihood. Variants with a minor allele frequency (MAF) ≥ 0.01 were classified as common and otherwise as rare. Exonic variants with potential functional effects were identified using the annotations in the database for nonsynonymous SNP functional predictions (dbNSFP, GRCh37/hg19 genome assembly). This filter uses SIFT, Provean, PolyPhen-2, Mutation Taster, Mutation Assessor, Gerp^++^, and PhyloP to predict a variant’s deleterious effect and is fully implemented in the SVS 8.3.3 Variant Classification module. Additionally, we also investigated the presence of variants associated with clinical disorders and annotated them according to the last report of ClinVar. Please see Figure 1.

GWAS Analysis: As mentioned in the introduction, given the low prevalence of ME we used 323 individuals belonging to Caucasian communities recruited and genotyped by the 1000 Genome Project (1 K Genomes) as controls [40]: Utah residents (CEPH) with Northern and Western European Ancestry (CEU, n = 32), Finnish individuals in Finland (FIN, n = 93), British individuals in England and Scotland (GBR, n = 86), an Iberian population in Spain (IBS, n = 14), and individuals from Tuscany in Italy (TSI, n = 98). The potential bias was minimal given the rareness of the ME phenotype. Furthermore, we estimated the index of fixation, better known as the *Fst* statistic, to evaluate the potential presence of microdifferentiation among the European populations, both as single units and combined as the “control” group, and our Australian ME/CFS cohort. For that estimation we used 18,818 variants that were excluded from the final association analysis (Figure 1). These 18,818 variants were common and polymorphic variants that met all quality control criteria and were pruned as redundant variations from the set of variants used for the final analysis through LD (window size: 50 markers; window increment: 5 markers, LD statistic: r^2^; r^2^ threshold: 0.5, LD method: CHM; inactivating 193,576 markers). The Fst estimated between the case and control groups was 0.04 (Standard Deviation = 0.05), defining no significant differences with an Fst = 0.0. As suggested by many authors, values of Fst below 0.05 were taken to be indicative of population homogeneity, and therefore an absence of microdifferentiation, suggesting that the case and control cohorts are homogeneous populations.

We studied the association of common polymorphic variants (MAF ≥ 0.01) to ME using single- and multi-locus linear mixed-effect models (SLMM and MLMM, respectively) with up to 10 steps in their backward/forward optimisation algorithms [41,42]. The advantage of these models was their inclusion of both fixed (genotype markers and covariates of any type) and random effects (family or population structure), the latter being used to account for potential inbreeding by including a kinship matrix (that is, the identity-by-descent [IBD] matrix, which in our case was estimated between all pairs of individuals using markers excluded from the final analysis after linkage disequilibrium [LD] pruning). An SLMM assumes that all loci have a small effect on the trait (simulating a multifactorial model), while an MLMM assumes that several interacting loci have a large effect (non-linear epistatic effects) [41,42].

Both types of models were implemented in SVS 8.3.3. The optimal model was selected using a comprehensive exploration of multiple criteria including the Extended Bayes Information Criteria (eBIC)*,* the Modified Bayes Information Criteria (mBIC)*,* and the Multiple Posterior Probability of Association (mPPA). After the estimation process using the forward/backward algorithm was finished, the coefficients ${\hat{\beta }}_{1},{\hat{\beta }}_{2},\ldots ,{\hat{\beta }}_{m}$ were extracted and a hypothesis test of the form *H*_0,*i*_:$\;\beta_{i}=0$ vs. *H*_1,*i*_: $\;\beta_{i}\neq 0$ was performed for the *ith* genetic variant to obtain the corresponding *p*-value (*i* = 1,2,…,*m*). Thus, the collection *P*_1_, *P*_2_, …, *P_m_* of *p*-values was subsequently corrected for multiple testing using the false discovery rate (FDR) [43]. Because the hypothesis tests being performed were of the same type, this correction needed to be performed on the resulting *m* *p*-values only. Given the complexity of patterns of transmission, and as this research forms part of an exploratory enterprise, we modelled different forms of transmission and maximised models following additive, dominant, and recessive inheritance. To evaluate the effect of covariates, we included age and gender as interacting factors and evaluated by direct inspection if any significant interaction was taking place.

Replication of Associated Variants in a Separate Cohort: Approval was sought from the NIH, and subsequently granted, allowing us to access and download raw genotype data collected from an ME/CFS cohort located in Nevada, the United States (dbGaP-phs001015.v1.p1) [22]. Affymetrix files for the 80 individuals who participated in the study were imported into Golden Helix^®^’s SVS 8.8.3 software. We applied the same protocol of quality control and filtering as was applied to our Australian ME/CFS cohort. Pruning of common, polymorphic, and redundant (LD criteria) data rendered 189,572 markers to build the IBD matrix for linear mixed-effect model maximisation. With the panel of significantly associated genes in the Australian ME/CFS cohort, we selected variants that were harboured within the coding region of the gene and the flanking 1000 bp. A total of 2488 genetic variants remained to be tested and were subject to the same set of analyses as previously described for the Australian ME/CFS cohort, considering the same set of SLMMs and MLMMs with different modes of transmission. To ensure the accurate detection of gene candidates, only replications with an FDR *p*-value of less than 0.05 (*p* < 0.05) were considered.

Pathway and Network Analyses: To identify over-representation of key ontogenetic functional network processes, i.e., cellular, biochemical, and physiological processes involving those genes harbouring the ME/CFS-associated common variants, we performed network and pathway enrichment analyses with different strategies of clustering exploration, namely, (i) single enrichment to detect the significant over-representation of functional annotations in one gene set (usually a group of genes which are significant in a given test) compared to another gene set (the rest of genes in the genome, for example); (ii) the heuristic interpretation of maps, networks, rich ontologies for diseases based on the biological role of candidate genes. For these analyses we used the modules FatiGO and SNOW implemented in Babelomics5.0^®^ [44,45,46,47], and the comprehensive set of modules implemented in Enrichr^®^ [48,49], allowing us to search for enrichments in all categories at once: Transcription, Pathways, Ontology, Disease/Drugs, and Cell Types, among others. Correction for multiple comparisons and the inclusion of nodes with direct physical interactions between the encoded proteins in the database were used to control for type I errors.

## 3. Results

ME/CFS cohort: The median age of the study participants was 48.0 years (CI: 39.3–56.0), with a sex ratio of 4.7:1.0, female to male (*n* = 80). All the participants were known ME/CFS patients with symptom lengths of between 2 and >20 years. This ME/CFS cohort has been described previously and compared to a non-ME/CFS (healthy) control group in relation to pathology markers and serum activin B, with symptom severity assessed via Weighted Standing Time (WST) [29,30]. DNA was collected from 77 of the 80 initially recruited ME/CFS participants (3 ME/CFS participants either did not provide consent, did not attend sample collection, and/or did not meet the inclusion criteria). An insufficient number of healthy controls consented to participation in the genetic study (<10), with comparisons subsequently made via the 1000 Genomes database.

Genetic analysis: DNA from 77 ME/CFS patients was subject to whole-exome capture, amplification, and sequencing. We identified a total of 4,792,938 variants (SNPs and Ins/Del), with 1,138,723 novel and 3,654,215 known rare and common variants, 3,368,071 markers with two alleles, 285,284 markers with one allele, and 860 markers with more than two alleles—Figure 1. We filtered out variants by applying criteria for call rate, number of alleles, heterozygosity, MAF, and HWE (*p*-value = 6.18 × 10^−7^), as described in the Materials and Methods Section and presented in Figure 1. A total of 470,903 variants remained for analysis. To avoid information redundancy, we pruned markers through LD following the criteria described in the Materials and Methods Section.

A total of 277,327 common variants remained for posterior analysis. These remaining variants were merged with 1000 Genomes Project data, and 100,148 common markers matched between both data sets. The LD redundant common variation from the 1000 Genomes Project (18,849 variants) was merged with the redundant variation from our Australian sample to build an IBD matrix that was used to maximise the mixed models. A total of 81,297 markers were used in the final association analysis (Figure 1).

**Figure 2:**
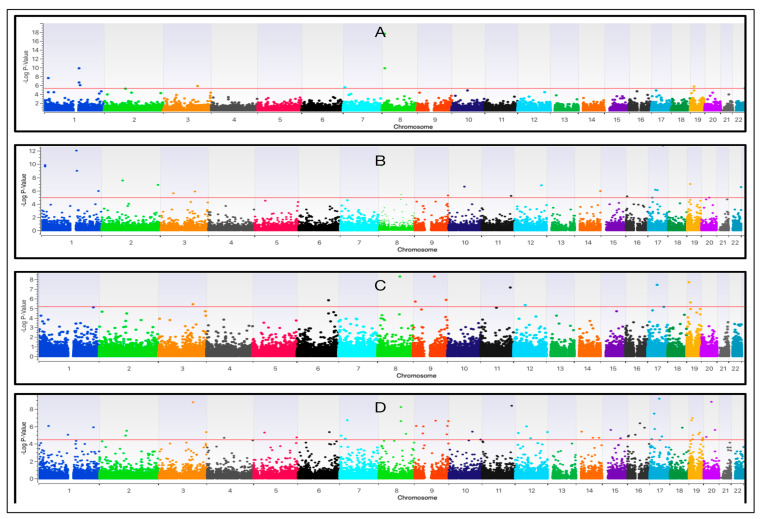
The Genome-Wide Association (GWA) analysis of ME patients (n = 77) recruited via the CFS Discovery Clinic (Donvale, Australia) and diagnosed using the ICC (5) prior to study entry. GWA Manhattan plots summarising the multi-locus linear mixed-effect model (MLMM) detection of top markers by (**A**) −log *p*-values and (**B**) false discovery rate (FDR)-calculated *p*-values. The figure also shows the results from the single-locus linear mixed-effect model (SLMM) analysis, reporting (**C**) −log *p*-values and (**D**) *p*-values calculated via FDR criteria (as performed for MLMM).

Manhattan plots depicting the genome significances reached after the maximisation of the two MLMMs (additive and dominant) and two SLMMs (additive and dominant) are presented in Figure 2A–D and Table 1.

The additive MLMM maximised with 10 variants (Table 1A), 4 of them harboured in genes that belong to the Neuroblastoma Breakpoint Family of proteins, namely, *NBPF1*, *NBPF10* (two variants—synonymous and intron)*,* and *NBPF16*. The signal with the strongest significance value for the additive MLMM was observed for a variant harboured in an intergenic (noncoding) region close to the coding region of the *FAM86B2* gene (rs2980473 and rs80169473). Another four significant variants were anchored to the intronic regions of the *serine/threonine kinase (ATR)*, *radial spoke head 10 homologue (RSPH10B)*, and *adhesion G protein-coupled receptor E5 - CD97 antigen (ADGRE5-CD97)*, and to the coding region of the *acrosin (ACR)* genes.

The additive SLMM maximized with 22 variants (Table 1B), some of them already associated with the MLMM, e.g., *NBPF1 NBPF10 NBPF16*, *ATR*, and *ADGRE5-CD97.* The SLMM’s strongest significance signal was observed for the *NBPF10* gene (rs1553120233, βreg = 0.01). Other genes implicated by this model (some harbouring variants with functional effect) were *endoplasmic reticulum oxidoreductase 1 beta (ERO1B)*, *CD8b2 molecule (CD8B*, SNP rs4514875, stop-retained variant*)*, *alkaline phosphatase*, *germ cell (ALPG*, SNP rs183793479, missense variant*)*, *Scm* like with four *mbt* domains *(SFMBT1)*, *LOC100506990, phosphatidylinositol-4,5-bisphosphate 4-phosphatase 2-Transmembrane Protein 55A (PIP4P2-TMEM55A)*, *G protein signalling modulator 1 (GPSM1)*, *receptor accessory protein 3 (REEP3)*, *sortilin related receptor 1 (SORL1)*, *ataxin 2 (ATXN2)*, *centrosomal protein 170B (CEP170B)*, *CREB binding protein (CREBBP)*, *envoplakin like (EVPLL)*, *leucine rich repeat containing 37B (LRRC37B)*, *keratin 24 (KRT24)*, and the *CDK5 regulatory subunit associated protein 1 (CDK5RAP1).*

The dominant MLMM maximised with 12 variants (Table 1C). Only variants in the *ATR*, *SORL1*, *KRT24*, and *PIP4P2-TMEM55A* genes were maximised by additive and dominant transmission. The strongest significance signal in the dominant MLMM was observed for the variant harboured in the *PIP4P2-TMEM55A* gene (rs13277356). A similar significance value was reached for the variant in the *neurotrophic receptor tyrosine kinase 2 (NTRK2) gene* (rs1659400). Other variants dominantly associated with ME/CFS were *laminin subunit alpha 2 (LAMA2)*, *protein tyrosine phosphatase receptor type D (PTPRD)*, *REX4 homologue*, *3′*-*5′ exonuclease (REXO4)*, *solute carrier family 38 member 2 (SLC38A2)*, *arylsulfatase G (ARSG)*, *zinc finger protein 266 (ZNF266)*, and *cytochrome P450 family 4 subfamily F member 8 (CYP4F8).*

The dominant SLML maximised with 58 variants (Table 1D). Several were included previously by the maximisation of other transmission models, and other genes with significantly associated variants included the following: the *Rap guanine nucleotide exchange factor 5 (RAPGEF5)*, *CUB and Sushi multiple domains 3 (CSMD3)*, *DCC netrin 1 receptor (DCC)*, *aldehyde dehydrogenase 18 family member A1 (ALDH18A1)*, *polypeptide N-acetylgalactosaminyltransferase 16 (GALNT16)*, *unc-79 homologue*, *NALCN channel complex subunit (UNC79)*, and *nuclear receptor coactivator 3 (NCOA3)* genes.

Enrichment analyses were also conducted to support these investigations, with a summary of the results presented in Table S1.

Comparison with Nevada (USA) ME/CFS sample: We conducted a replication study of our positive Australian associations on the GWAS raw data genotyped in an ME/CFS cohort recruited from Nevada in the United States [22]. Several associations shared by both cohorts were successfully identified, namely (1) a cluster harboured in the genomic region encoding *protein tyrosine phosphatase receptor type D (PTPRD)* (Australian cohort, *p*-value = 2.21 × 10^−6^; Schlauch et al. cohort, *p*-value = 1.14 × 10^−6^); (2) a cluster of six markers harboured in the *CUB* and *Sushi multiple domains 3 (CSMD3*); (3) other variants anchored in the coding regions of the *RAPGEF5*, *CSMD3*, *DCC*, *ALDH18A1*, *GALNT16*, *UNC79*, *NCOA3* genes. We found novel and functional variants harboured in the coding regions of some of these genes, suggesting that these mutations might underlie the common association highlighted inside of these genome regions.

## 4. Discussion

We have identified common genome variants associated with ME/CFS susceptibility through the capture and sequencing of patients’ whole-exomes. The ME/CFS patients’ exome genotypes were contrasted with those reported by the 1 K Genomes Project data for ethnically and genetically matched, genetically homogeneous populations. Following this analysis, we replicated several significantly associated signals in a disparate population, identified by a previously published case–control cohort [22]. The associated genes clustered in several processes related to brain evolution, neurogenesis, and neurodifferentiation, with remarkable implications in neurological neoplasia, such as neuroblastoma and neuropsychiatric disorders (e.g., schizophrenia, autism), as well as developmental and neurodegenerative disorders. An unanticipated result was the connection to the Olduvai genomic region [24], which has been previously detected in human neurological disorders, like those listed above.

According to the ICC, ME (CFS) “… is an acquired neurological disease with complex global dysfunctions,” with at least one symptom from three of four *Neurological Impairment* categories required to fulfil a diagnosis (e.g., information processing, sleep difficulties, vision focus, memory loss) [5]. A number of magnetic resonance imaging (MRI), magnetic resonance spectroscopy (MRS) and functional magnetic resonance imaging (fMRI) investigations have been performed to detect brain structure and function differences between ME/CFS patients and control participants, sometimes with correlations to cognitive performance and/or clinical features emphasised [50,51,52]. These studies’ observations included statistically significant brain metabolite and temperature differences, suggesting neuroinflammation, differences in white matter, grey matter, and brain stem volumes, and variable myelination patterns. Systematic and scoping reviews have been performed to add clarity to these findings, exploring the consistency of ME/CFS-associated neuro-abnormalities [53,54,55].

For the Australian cohort investigated, the genes implicated in conferring ME/CFS susceptibility included the Neuroblastoma Breakpoint Family members *NBPF1* (rs3897177), *NBPF10* (rs1553120233), and *NBPF16* (rs200632836). Other significantly associated variants were detected in *serine/threonine kinase (ATR)*, the *radial spoke head 10 homologue (RSPH10B)*, the *adhesion G protein-coupled receptor E5-CD97 antigen (ADGRE5-CD97),* and the *neurotrophic receptor tyrosine kinase 2 (NTRK2)*, among others (Table 1). We demonstrated, by population genetics analysis, that there was no microdifferentiation among cohorts, and therefore that this comparison of genetic distributions was appropriate for the mixed models utilised [40].

Replication from the Nevada cohort of the significant associations between genomic variants and ME/CFS susceptibility found in the Australian analysis was successful for several genes, including *PTPRD*, *RAPGEF5*, *CSMD3*, *DCC*, *ALDH18A1*, *GALNT16*, *UNC79*, *NCOA3*. Interestingly, several variants were noticeable in the *CSMD3*, *PTPRD*, and *DCC* genes (Table 1E–G). Also, the comparison with the Australian results showed agreement in relation to the primacy of neurological genes, with *GRIK* and *NPAS2* being significant [22], repeating observations from an earlier GWAS, supported by gene expression results [56]. *GRIK2* has also been linked to schizophrenia and autism, while *NPAS2* is associated with circadian rhythm control [22,56].

Given the very low prevalence of ME/CFS, we hypothesised that highly penetrant genetic variants of major effect underpin susceptibility and, therefore, population frequency [57]. On this basis it was reasonable to compare genetic variation in the Australian cohort with the standard distributions of genetic variants estimated from large cohorts, without considering particular diseases as criteria for ascertainment. This was achieved by reference to the 1000 Genomes Project, which serves as global reference for human genetic variation [39]. In the past, we, and others, have successfully used this strategy and mapped major genes underpinning susceptibility to diseases of low and moderate prevalence such as membranous nephropathy [58], glioma [59], and epithelial ovarian cancer [60], among others [61].

Three of the leading genome regions associated with ME/CFS harbour gene members of the NBPF, namely *NBPF1*, *NBPF10*, and *NBPF16.* As indicated by several reports, the NBPF is a cluster of genes spanning disparate regions of human chromosome 1 and was generated by duplications in primate evolution. The presence of significant NBPF signals, found in the Australian ME/CFS cohort, were unlikely to be spurious associations. The three primary genes are separated by vast chromosomal distances and exist in full linkage equilibrium. Furthermore, the contrasting size length of the *NBPF1*, *NBPF10*, and *NBPF16* coding regions suggest that the possibility of spurious associations related to gene length are unlikely.

The NBPF was originally identified by the disruption of *NBPF1* in a neuroblastoma patient [62], and so it may play a role in neuroblastoma and other cancers [63,64,65]. It has also been identified as an important contributor to human brain evolution [63,66]. *NBPF1* consists of 22 genes and pseudogenes with numerous low-copy repetitive elements and high intergenic and intragenic sequence identity in their coding and noncoding regions [63]. Expression studies in neuroblastoma and colon cancer showed that cancer cell decreases in clonal expansion were linked to the NBPF, suggesting that NBPF1 can act as tumour suppressor [64,65].

Particularly fascinating to note for the NBPF genes is the location of variations in the Olduvai genomic region, so named in recognition of fundamental 19th–20th century research conducted in the African location of the same name, which provided seminal observations into the human evolution that differentiates humans from other primates, and as such captures the essence of human biology [24]. The NBPF encodes most of the human-specific copies of Olduvai (119 copies from a total of 165), and sequences encoding Olduvai (DUF1220) protein domains show the largest human-specific increase in copy number of any coding region in the genome, which has been linked to human brain evolution (~300 total copies, of which ~165 are human-specific).

*NBPF10* and *NBPF16* are adjacent to three human-specific *NOTCH2NL* genes that promote cortical neurogenesis [67,68]. Phylogenetic and transcriptomic evidence strongly suggest that these *NOTCH2NL*/*NBPF* gene pairs evolved jointly, as two-gene units, very recently in human evolution, and are likely co-regulated. Olduvai domains and adjacent *NOTCH2NL* genes may function in a coordinated, complementary fashion to promote neurogenesis and human brain expansion in a dosage-related manner [67,68], and promote proliferation in neural stem cells [69]. Interestingly, variation in Olduvai copy number has been associated with cognitive disease, autism, schizophrenia, microcephaly, and macrocephaly [23,70,71,72,73]. This trade-off between promoting brain expansion and deleterious effects suggests that the Olduvai family is an active target of evolution specific to the human lineage. In the context of copy-number variation (CNV), a future ME/CFS investigation is recommended to address questions of dosage, as found in autism and other cognitive diseases [70,71,72,73], and thereby extend the results obtained herein via exome capture that emphasise this region. Once the genetics are confirmed, functional studies focused on the identified ME/CFS-associated genes will be required to validate disease phenotype.

Supporting the NBPF association, the enrichment analysis showed the over-representation of GO biological process Regulation of Dendrite Development (GO:0050773) clustering in the *DBN1*, *PTPRD*, *DCC*, and *NTRK2* genes. The *PTPRD* gene encodes a member of the protein tyrosine phosphatase (PTP) family, signalling molecules associated with cellular processes including cell growth, differentiation, the mitotic cycle, and oncogenic transformation. Members of the PTP family also promote neurite growth and regulate neurons’ axon guidance. *PTPRD* is one of the most frequently inactivated genes across human cancers, including glioblastoma multiforme (GBM) [74]. It is well known that pre-synaptic PTPRD promotes the differentiation of glutamatergic synapses, and several studies link *PTPRD* genetic variation to psychiatric phenotypes such as schizophrenia, bipolar disorder and mood instability [75], obsessive–compulsive disorder [76,77], and weight gain with antipsychotic medication [78]. Variants harboured in PTRPD have also been associated with susceptibility to the de-development of neurofibrillary tangles [79].

The significance of the *ALDH18A1* gene, a member of the aldehyde dehydrogenase family, provides support for several observations on metabolomic profile imbalance in ME/CFS patients [80,81], including in patients recruited for research via the same clinic as this study [82,83]. *ALDH18A1* encodes a bifunctional ATP- and NADPH-dependent mitochondrial enzyme with both gamma-glutamyl kinase/phosphate reductase activities, and is critical to the de novo biosynthesis of proline, ornithine, and arginine. The intermediate compound, pyrroline-5-carboxylate, was previously observed as being increased in plasma from female and male ME/CFS patients, among several metabolic perturbations [80].

The significance of *ALDH18A1*, in addition to its role in metabolic perturbations, draws attention to mitochondrial function in ME/CFS patients. While future functional studies on the myriad neurodevelopment genes are needed to confirm their significance, mitochondrial function studies have been conducted on the same patient cohort as presented here [84]. Notable for ME/CFS dysfunction were Complex V insufficiency combined with TORC-1 increases in comparison to healthy (non-ME) control participants. Whether *ALDH18A1* is directly involved in this ME/CFS mitochondrial function profile will require further investigation.

Investigations of allelic associations that confer susceptibility to ME/CFS are challenging due to several genetic and technical factors, which this study looked to address and overcome. The problems include the low ME/CFS prevalence that prevents achieving the sample requirements for conducting a successful GWAS and using genotyping technology designed to characterise common and known allelic variations, rather than rare and novel variants, which are most likely for ME/CFS. Also, as detailed by Graboswka et al. [15], partial and heterogeneous quality control in the majority of the published genetic association studies will militate against replication comparisons, as well as discouraging data amalgamation. These issues are also limited by the poor understanding of ME/CFS pathophysiology [5].

The study was also limited by its relatively small sample size (*n* = 77). To address this limitation, all participants were examined by a medical practitioner (D.P.L.) with 15 years exclusive ME/CFS patient experience and via the ICC (of which D.P.L. was an author) [5], with the lead author (M.A.-B.) providing expertise in pooling/bootstrap GWAS analysis methods to further counter this issue [21].

We found whole-exome significant association between genome variants and the susceptibility to develop ME/CFS, mostly configuring interactive oligogenic and/or multifactorial models of transmission. Intriguingly, these variants are harboured in genes associated with mental disorders—i.e., wide-spectrum autistic syndrome, mood disorders, depression, and schizophrenia—as well as with the development of neoplasia and neurological conditions.

## Figures and Tables

**Table 1:** Top-ranked genes and detected sequence ontologies characterised by exome capture analyses (multi-locus and single-locus models) of ME participants (n = 77) diagnosed via International Consensus Criteria (5).

A. Multi-Locus Additive Model										
Chr	Position	Identifier	Related Gene *	Sequence Ontology (Combined)	Ref/Alt	MAF	*p*-Value	Beta (βreg)	Beta SE	FDR
1	16909052	rs3897177	NBPF1	synonymous	C/T	0.36	3.15 × 10^−8^	0.055	0.01	6.36 × 10^−4^
1	145303971	rs10910794	NBPF10	synonymous	A/G	0.24	2.63 × 10^−7^	0.047	0.009	4.25 × 10^−3^
1	145355624	rs1553120233	NBPF10	intron	C/T	0.5	1.81 × 10^−10^	0.08	0.012	7.33 × 10^−6^
1	148756363	rs200632836	NBPF16	intergenic	A/G	0.45	1.04 × 10^−6^	0.047	0.01	0.01
3	142233470	rs6440086	ATR	intron	T/C	0.49	1.68 × 10^−6^	−0.038	0.008	0.02
7	6006431	rs2711192	RSPH10B	intron	G/A	0.45	3.73 × 10^−6^	−0.038	0.008	0.03
8	12291415	rs80169473	FAM86B2	intergenic	C/A	0.48	1.98 × 10^−10^	−0.052	0.008	5.32 × 10^−6^
8	12294359	rs2980473	FAM86B2	intergenic	G/C	0.35	2.62 × 10^−18^	0.096	0.01	2.11 × 10^−13^
19	14499357	rs2302094	ADGRE5-CD97	intron	T/A	0.05	2.48 × 10^−6^	0.085	0.018	0.03
22	51183255	rs5771002	ACR	missense	A/G	0.48	6.09 × 10^−6^	0.037	0.008	0.05
**B. Single-Locus Additive Model**										
**Chr**	**Position**	**Identifier**	Related Gene *	**Sequence** **Ontology** **(Combined)**	**Ref/Alt**	**MAF**	***p*** **-Value**	**Beta (βreg)**	**Beta SE**	**FDR**
1	16904121	rs5003678	NBPF1	intron	T/G	0.41	1.61 × 10^−10^	0.08	0.012	6.52 × 10^−6^
1	16909052	rs3897177	NBPF1	synonymous	C/T	0.36	2.43 × 10^−10^	0.07	0.011	4.91 × 10^−6^
1	145355624	rs1553120233	NBPF10	intron	C/T	0.5	9.26 × 10^−13^	0.1	0.014	7.49 × 10^−8^
1	148756363	rs200632836	NBPF16	intergenic	A/G	0.45	1.09 × 10^−9^	0.07	0.011	1.76 × 10^−5^
1	236396811	rs2463185	ERO1B	intron	C/A	0.49	1.20 × 10^−6^	0.05	0.01	6.91 × 10^−3^
2	87069431	rs4514875	CD8B	stop_retained	C/T	0.47	3.06 × 10^−8^	0.06	0.01	4.13 × 10^−4^
2	233273504	rs183793479	ALPG	missense	T/G	0.49	1.36 × 10^−7^	0.05	0.01	1.37 × 10^−3^
3	52965713	rs2710339	SFMBT1	intron	G/A	0.47	2.75 × 10^−6^	0.04	0.009	0.01
3	142233470	rs6440086	ATR	intron	T/C	0.49	1.44 × 10^−6^	−0.05	0.009	7.27 × 10^−3^
8	12294359	rs2980473	LOC100506990	intergenic	G/C	0.35	1.93 × 10^−10^	0.07	0.011	5.19 × 10^−6^
8	92052619	rs13277356	PIP4P2- TMEM55A	intron	C/A	0.48	3.90 × 10^−6^	−0.05	0.01	0.02
9	139249991	rs28603210	GPSM1	intron	T/C	0.43	5.17 × 10^−6^	0.04	0.009	0.02
10	65355538	rs10733794	REEP3	intron	A/G	0.46	2.57 × 10^−7^	0.05	0.01	2.08 × 10^−3^
11	121459522	rs1792122	SORL1	intron	C/T	0.5	6.73 × 10^−6^	−0.04	0.009	0.03
12	112036797	rs4098854	ATXN2	synonymous	C/T	0.38	1.79 × 10^−7^	0.05	0.01	1.61 × 10^−3^
14	105356241	rs61996002	CEP170B	intron	A/G	0.4	1.23 × 10^−6^	0.05	0.009	6.62 × 10^−3^
16	3791261	rs129968	CREBBP	intron	A/G	0.49	8.04 × 10^−6^	0.04	0.01	0.03
17	18290687	rs35418981	EVPLL	intron	T/A	0.45	1.17 × 10^−5^	−0.04	0.01	0.04
17	30358510	rs10752705	LRRC37B	intron	G/A	0.46	7.53 × 10^−7^	0.05	0.009	5.07 × 10^−3^
17	38858029	rs6416908	KRT24	intron	A/T	0.45	8.56 × 10^−7^	−0.05	0.01	5.32 × 10^−3^
19	14499357	rs2302094	ADGRE5	intron	T/A	0.05	1.11 × 10^−7^	0.12	0.021	1.28 × 10^−3^
20	31945861	rs291670	CDK5RAP1	intergenic	G/A	0.5	1.21 × 10^−5^	−0.04	0.009	0.04
22	51183255	rs5771002	ACR	missense	A/G	0.48	3.07 × 10^−7^	0.05	0.01	2.26 × 10^−3^
**C. Multi-Locus Dominant Model**										
**Chr**	**Position**	**Identifier**	Related Gene *	**Sequence** **Ontology** **(Combined)**	**Ref/Alt**	**MAF**	***p*** **-Value**	**Beta (βreg)**	**Beta SE**	**FDR**
3	142233470	rs6440086	ATR	intron	T/C	0.49	3.88 × 10^−6^	−0.068	0.015	0.03
6	129622257	rs3798664	LAMA2	intron	A/G	0.37	1.51 × 10^−6^	−0.058	0.012	0.02
8	92052619	rs13277356	PIP4P2- TMEM55A	intron	C/A	0.48	5.30 × 10^−9^	−0.088	0.015	4.28 × 10^−4^
9	8436361	rs7854171	PTPRD	intron	A/G	0.45	2.21 × 10^−6^	−0.062	0.013	0.02
9	87325994	rs1659400	NTRK2	intron	A/G	0.46	5.77 × 10^−9^	−0.082	0.014	2.33 × 10^−4^
9	136274058	rs7030175	REXO4	intron	G/T	0.46	1.44 × 10^−6^	−0.064	0.013	0.02
11	121459522	rs1792122	SORL1	intron	C/T	0.5	7.81 × 10^−8^	−0.081	0.015	1.26 × 10^−3^
12	46761324	rs1873793	SLC38A2	intron	C/T	0.49	5.20 × 10^−6^	−0.06	0.013	0.04
17	38858029	rs6416908	KRT24	intron	A/T	0.45	4.18 × 10^−8^	−0.081	0.014	8.45 × 10^−4^
17	66303352	rs2072268	ARSG	5_primeUTR	G/A	0.39	7.35 × 10^−6^	−0.053	0.012	0.05
19	9526017	rs7258150	ZNF266	intron	G/A	0.44	2.12 × 10^−8^	−0.076	0.013	5.71 × 10^−4^
19	15728556	rs4019755	CYP4F8	intron	A/G	0.42	2.68 × 10^−6^	−0.059	0.012	0.02
**D. Single-Locus Dominant Model**										
**Chr**	**Position**	**Identifier**	Related Gene *	**Sequence** **Ontology** **(Combined)**	**Ref/Alt**	**MAF**	***p*** **-Value**	**Beta (βreg)**	**Beta SE**	**FDR**
1	40234765	rs230319	BMP8B	intron	A/G	0.42	9.03 × 10^−7^	−0.074	0.015	5.21 × 10^−3^
1	120301432	rs1441010	HMGCS2	intron	A/G	0.44	9.26 × 10^−6^	−0.070	0.016	0.02
1	225555856	rs12756111	DNAH14	intron	C/T	0.48	1.28 × 10^−6^	−0.080	0.016	5.46 × 10^−3^
2	108443647	rs78477381	RGPD4	intron	C/G	0.38	1.23 × 10^−5^	−0.066	0.015	0.02
2	112870730	rs7581849	PIP4P2- TMEM87B	intron	G/A	0.49	3.38 × 10^−6^	−0.077	0.016	0.01
3	142233470	rs6440086	ATR	intron	T/C	0.49	1.70 × 10^−9^	−0.105	0.017	4.59 × 10^−5^
3	195460955	rs1808432	MUC20	intergenic	T/A	0.34	4.64 × 10^−6^	−0.068	0.015	0.01
4	70596977	rs7660770	SULT1B1	intron	G/A	0.42	2.19 × 10^−5^	−0.063	0.015	0.03
5	43614968	rs4991951	NNT	intron	A/G	0.46	5.57 × 10^−6^	−0.072	0.016	0.01
5	176898619	rs335420	DBN1	intron	T/C	0.42	1.85 × 10^−5^	−0.062	0.014	0.03
6	130379160	rs12661232	L3MBTL3	intron	T/C	0.43	4.57 × 10^−6^	−0.073	0.016	0.01
7	6006431	rs2711192	RSPH10B	intron	G/A	0.45	1.21 × 10^−5^	−0.069	0.016	0.02
7	22184167	rs1859806	RAPGEF5	intron	G/A	0.42	2.80 × 10^−5^	−0.063	0.015	0.04
7	30537640	rs4720005	GGCT	intron	G/T	0.47	2.09 × 10^−7^	−0.087	0.017	1.88 × 10^−3^
8	90802099	rs400411	RIPK2	intron	A/G	0.50	2.51 × 10^−7^	−0.085	0.016	1.84 × 10^−3^
8	92052619	rs13277356	PIP4P2- TMEM55A	intron	C/A	0.48	6.12 × 10^−9^	−0.101	0.017	9.89 × 10^−5^
8	113650725	rs7833307	CSMD3	intron	C/T	0.37	7.08 × 10^−6^	−0.066	0.014	0.02
9	8436361	rs7854171	PTPRD	intron	A/G	0.45	9.14 × 10^−7^	−0.077	0.016	4.93 × 10^−3^
9	34724786	rs3739878	FAM205A	synonymous	G/A	0.40	6.51 × 10^−6^	−0.066	0.014	0.02
9	36121065	rs2149006	RECK	intron	C/G	0.49	1.04 × 10^−6^	−0.080	0.016	4.67 × 10^−3^
9	87325994	rs1659400	NTRK2	intron	A/G	0.46	2.24 × 10^−7^	−0.084	0.016	1.81 × 10^−3^
9	133916387	rs11244254	LAMC3	intron	G/T	0.38	8.83 × 10^−6^	−0.064	0.014	0.02
9	136274058	rs7030175	REXO4	intron	G/T	0.46	9.67 × 10^−7^	−0.078	0.016	4.88 × 10^−3^
9	139944588	rs7869655	ENTPD2	intron	T/C	0.46	2.70 × 10^−7^	−0.091	0.017	1.82 × 10^−3^
10	97367511	rs11188397	ALDH18A1	intron	C/A	0.41	4.29 × 10^−6^	−0.070	0.015	0.01
10	129908986	rs2782870	MKI67	intron	C/A	0.44	3.49 × 10^−5^	−0.062	0.015	0.05
11	121459522	rs1792122	SORL1	intron	C/T	0.50	4.43 × 10^−9^	−0.102	0.017	8.94 × 10^−5^
12	12879570	rs34322	APOLD1	intron	T/C	0.43	5.65 × 10^−6^	−0.071	0.015	0.01
12	46761324	rs1873793	SLC38A2	intron	C/T	0.49	1.02 × 10^−6^	−0.078	0.016	4.86 × 10^−3^
12	64001613	rs2202644	DPY19L2	intron	G/A	0.42	2.54 × 10^−5^	−0.064	0.015	0.04
12	133378852	rs10781650	GOLGA3	intron	T/C	0.47	4.63 × 10^−6^	−0.071	0.015	0.01
14	21970379	rs1263793	METTL3	intron	A/G	0.43	4.04 × 10^−6^	−0.072	0.015	0.01
14	69809143	rs1296214	GALNT16	intron	G/A	0.37	2.05 × 10^−5^	−0.060	0.014	0.03
14	94120712	rs55882426	UNC79	intron	C/T	0.42	2.02 × 10^−5^	−0.064	0.015	0.03
15	34639015	rs383086	NUTM1	intron	C/T	0.42	2.57 × 10^−6^	−0.073	0.015	8.66 × 10^−3^
15	74365264	rs1835371	GOLGA6A	intron	T/G	0.34	2.44 × 10^−5^	−0.059	0.014	0.04
16	2014954	rs2302176	SNHG9	intergenic	C/T	0.49	1.54 × 10^−5^	−0.070	0.016	0.03
16	5135380	rs6775	ALG1-EEF2KMT	3_prime_UTR	A/G	0.43	1.17 × 10^−5^	−0.067	0.015	0.02
16	31393544	rs9929832	ITGAX	3_prime_UTR	C/T	0.39	9.94 × 10^−6^	−0.064	0.014	0.02
16	50333837	rs8045659	ADCY7	intron	T/C	0.45	4.34 × 10^−7^	−0.080	0.016	2.70 × 10^−3^
16	69986839	rs2650542	CLEC18A	intron	G/C	0.44	1.53 × 10^−6^	−0.076	0.016	5.91 × 10^−3^
17	6537526	rs9914024	KIAA0753	intron	G/A	0.39	3.08 × 10^−5^	−0.061	0.014	0.04
17	18290687	rs35418981	EVPLL	intron	T/A	0.45	3.67 × 10^−8^	−0.089	0.016	4.94 × 10^−4^
17	20355058	rs4332792	LGALS9B	intron	T/G	0.41	2.04 × 10^−6^	−0.072	0.015	7.48 × 10^−3^
17	38858029	rs6416908	KRT24	intron	A/T	0.45	6.47 × 10^−10^	−0.105	0.017	5.23 × 10^−5^
17	49281678	rs28410310	MBTD1	intron	T/C	0.45	1.57 × 10^−5^	−0.068	0.016	0.03
18	50924132	rs11082992	DCC	intron	T/C	0.42	1.43 × 10^−6^	−0.075	0.015	5.78 × 10^−3^
19	9526017	rs7258150	ZNF266	intron	G/A	0.44	2.02 × 10^−7^	−0.082	0.015	2.04 × 10^−3^
19	14499357	rs2302094	ADGRE5	intron	T/A	0.05	1.11 × 10^−7^	0.115	0.021	1.28 × 10^−3^
19	14639947	rs7249458	DNAJB1	intron	A/T	0.37	3.09 × 10^−5^	−0.061	0.015	0.04
19	15728556	rs4019755	CYP4F8	intron	A/G	0.42	1.01 × 10^−5^	−0.067	0.015	0.02
19	40375967	rs62106959	FCGBP	intron	C/T	0.40	8.18 × 10^−6^	−0.068	0.015	0.02
19	44132559	rs8101721	CADM4	intron	G/C	0.42	5.00 × 10^−6^	−0.067	0.014	0.01
19	55773590	rs10403164	HSPBP1	3_prime_UTR	A/G	0.44	2.58 × 10^−5^	−0.064	0.015	0.04
19	56274506	rs147984855	RFPL4A	missense	G/A	0.11	2.04 × 10^−5^	0.070	0.016	0.03
20	10624926	rs6077861	JAG1	intron	A/T	0.36	1.60 × 10^−5^	−0.064	0.015	0.03
20	31945861	rs291670	CDK5RAP1	intergenic	G/A	0.50	1.58 × 10^−9^	−0.107	0.017	6.40 × 10^−5^
20	46270379	rs623953	NCOA3	intron	G/A	0.46	2.51 × 10^−6^	−0.074	0.016	8.82 × 10^−3^
**E. Multi-Locus Dominant Model**										
**Chr**	**Position**	**Identifier**	Related Gene *	**Sequence** **Ontology** **(Combined)**	**Ref/Alt**		***p*-Value**	**Beta (βreg)**	**Beta SE**	**FDR**
9	8318231	rs996924	PTPRD	intron	A/G		4.55 × 10^−11^	0.714	0.092	1.13 × 10^−7^
**F. Single-Locus Additive Model**										
**Chr**	**Position**	**Identifier**	Related Gene *	**Sequence** **Ontology** **(Combined)**	**Ref/Alt**		***p*-Value**	**Beta (βreg)**	**Beta SE**	**FDR**
7	22278040	rs11766861	RAPGEF5	Intron	A/T		7.96 × 10^−5^	0.350	0.08	4.94 × 10^−2^
8	114359441	rs17608734	CSMD3	intron	G/T		3.61 × 10^−5^	−0.320	0.073	3.00 × 10^−2^
9	8318231	rs996924	PTPRD	intron	A/G		1.14 × 10^−6^	0.483	0.091	2.84 × 10^−3^
18	50369520	rs1560521	DCC	intron	G/A/C		2.63 × 10^−5^	0.378	0.084	3.27 × 10^−2^
**G. Multi-Locus Recessive Model**										
**Chr**	**Position**	**Identifier**	**Related** Gene *	**Sequence** **Ontology** **(Combined)**	**Ref/Alt**		***p*-Value**	**Beta (βreg)**	**Beta SE**	**FDR**
8	113617156	rs4876478	CSMD3	intron	T/G		3.81 × 10^−13^	−0.917	0.1035	4.73 × 10^−10^
8	114359441	rs17608734	CSMD3	intron	G/T		2.11 × 10^−22^	−0.872	0.0618	5.25 × 10^−19^
8	114399612	rs4311682	CSMD3	intron	A/G		9.22 × 10^−5^	0.933	0.2251	1.53 × 10^−2^
8	114406336	rs4354335	CSMD3	intron	G/A		9.22 × 10^−5^	0.933	0.2251	1.43 × 10^−2^
8	114418955	rs7002354	CSMD3	intron	T/C		9.22 × 10^−5^	0.933	0.2251	1.35 × 10^−2^
8	114436474	rs2942852	CSMD3	intron	T/G		9.22 × 10^−5^	0.933	0.2251	1.27 × 10^−2^
9	8409888	rs3847293	PTPRD	intron	C/G		1.80 × 10^−7^	−0.917	0.1588	6.40 × 10^−5^
9	8845429	rs2570961	PTPRD	intron	G/A		1.80 × 10^−7^	−0.917	0.1588	1.12 × 10^−4^
9	8897215	rs7866753	PTPRD	intron	C/T		9.22 × 10^−5^	0.933	0.2251	1.21 × 10^−2^
9	8901739	rs10815990	PTPRD	intron	A/G		9.22 × 10^−5^	0.933	0.2251	1.15 × 10^−2^
9	9270379	rs12341573	PTPRD	intron	G/T		9.22 × 10^−5^	0.933	0.2251	1.09 × 10^−2^
9	9829690	rs1746813	PTPRD	intron	G/C		7.63 × 10^−6^	−0.938	0.1942	1.73 × 10^−3^
9	9904274	rs16930522	PTPRD	intron	G/A		7.63 × 10^−6^	−0.938	0.1942	1.90 × 10^−3^
9	10254793	rs2498611	PTPRD	intron	T/G		1.80 × 10^−7^	−0.917	0.1588	8.96 × 10^−5^
9	87533389	rs6559836	NTRK2	intron	G/A		7.63 × 10^−6^	−0.938	0.1942	2.37 × 10^−3^
9	87631034	rs2378672	NTRK2	intron	C/T		7.63 × 10^−6^	−0.938	0.1942	2.11 × 10^−3^
10	97392993	rs3750700	ALDH18A1	intron	T/C		4.75 × 10^−4^	−0.288	0.0785	4.37 × 10^−2^
14	69734498	rs1890939	GALNT16	intron	C/G		6.71 × 10^−12^	−0.772	0.0942	5.57 × 10^−9^
14	93902973	rs28385502	UNC79	intron	A/G		9.22 × 10^−5^	0.933	0.2251	1.04 × 10^−2^
18	50567129	rs11874663	DCC	intron	G/A		7.63 × 10^−6^	−0.938	0.1942	1.58 × 10^−3^
18	50597529	rs4995148	DCC	intron	T/A		7.63 × 10^−6^	−0.938	0.1942	1.46 × 10^−3^
18	50618359	rs7233997	DCC	intron	G/A		3.86 × 10^−4^	−0.620	0.1665	4.18 × 10^−2^
18	50622857	rs9957443	DCC	intron	T/G		3.86 × 10^−4^	−0.620	0.1665	4.00 × 10^−2^
18	50622885	rs16956110	DCC	intron	C/T		3.86 × 10^−4^	−0.620	0.1665	3.84 × 10^−2^
18	50623189	rs16956114	DCC	intron	G/A		7.63 × 10^−6^	−0.938	0.1942	1.36 × 10^−3^
18	50668321	rs9956477	DCC	intron	C/A		3.86 × 10^−4^	−0.620	0.1665	3.69 × 10^−2^
20	46215501	rs6066395	NCOA3	intron	G/A		1.80 × 10^−7^	−0.917	0.1588	7.47 × 10^−5^

Chr: Chromosome; Seq-Ontology: Sequence Ontology; Ref/Alt: Reference and Alternate Alleles; MAF: Minor Allele Frequency; Beta (βreg): Regression Beta; Beta SE: Beta Standard Error; FDR: False Discovery Rate. * For intergenic variants, the annotated gene is the closest one to the variant.

## Data Availability

The reference data set used for the analyses described in this manuscript was obtained from dbGaP at https://dbgap.ncbi.nlm.nih.gov/beta/home through dbGaP study accession number phs001015.v1.p1. Data from the *New Strategies to Decipher the Pathophysiology of Chronic Fatigue Syndrome* project were provided by Vincent Lombardi on behalf of his collaborators at the University of Nevada, Reno. Vincent Lombardi and his collaborators request that publications resulting from these data cite their original publication (see Reference [22]). Sequence data are available by request to M. Arcos-Burgos (from recognised research groups who have complied with the relevant ethical criteria defined by our institutions and clinical committees).

## Supplementary Materials

The following supporting information can be downloaded at: https://www.mdpi.com/article/10.3390/diagnostics15121542/s1, Table S1: Enrichment analysis results from exomes investigated for ME/CFS patients confirmed by the ICC (Melbourne region, Victoria, Australia); Figure S1: Average sequencing depth (bar plot) and coverage (dot-line plot) in each chromosome investigated (exome capture) from Australian ME/CFS patients (*n* = 77) assessed and confirmed via the International Consensus Criteria (ICC); Figure S2: High resolution of SNPs and CNVs spanning the NBPF1 gene from the Australian ME/CFS patient cohort investigated post exome capture; Figure S3: Coverage, depth and pile up of readings spanning NBPF1, with a zoom in over the last 6 exons of the gene at the 3’ region detected in the investigated Australian ME/CFS cohort.
