# Supplementary material for: Neurodevelopment Genes Encoding Olduvai Domains Link Myalgic Encephalomyelitis to Neuropsychiatric Disorders
Source: Diagnostics (Basel). 2025 Jun 17;15(12):1542. doi: 10.3390/diagnostics15121542 (PMC12191824; doi:10.3390/diagnostics15121542)
Supplement: Supplementary file 1 [file diagnostics-15-01542-s001.zip › Suppl_Enrich.Table.pdf]

Table S1 - Enrichment analysis results from exomes investigated for ME/CFS patients confirmed by the ICC* (Melbourne region, Victoria, Australia).

Term or GO biological process propagated	Term Size	Term Size - In Genome	List1 Positives	List1 Negatives	List1 %	List2 Positives	List2 Negatives	List2 %	List1 Positives IDs	List2 Positive IDs	Odds Ratio (log e)	P-value	Corrected P-value
Mbt (IPR004092)	34	44	3	73	3.95	31	208322	0.01	SFMBT1, L3MBTL3, MBTD1	ENST00000422861, ENST00000373135, ENST00000373134, ENST00000251900, ENST00000400105, ENST00000400104, ENST00000372595, ENST00000372596, ENST00000372597, ENST00000216237, ENST00000456518, ENST00000452106, ENST00000583809, ENST00000317931, ENST00000284898, ENST00000418998, ENST00000449635, ENST00000445228, ENST00000326197, ENST00000379711, ENST00000379713, ENST00000427442, ENST00000402904, ENST00000361972, ENST00000337495, ENST00000361705, ENST00000397167, ENST00000397171, ENST00000397174, ENST00000361191, ENST00000583054	5.62	2.76 E-7	1.20 E-3

* ICC (International Consensus Criteria - Reference 5).

GO biological process propagated	Term Size	Term Size - In Genome	List1 Positives	List1 Negatives	List1 %	List2 Positives	List2 Negatives	List2 %	List1 Positives IDs	List2 Positive IDs	Odds Ratio (log e)	P-value	Corrected P-value
Regulation of dendrite development (GO: 0050773)	192	200	4	72	5.26	188	208165	0.09	DBN1, PTPRD, DCC, NTRK2	ENST00000422704, ENST00000350811, ENST00000298159, ENST00000343529, ENST00000615540, ENST00000252486, ENST00000428317, ENST00000373873, ENST00000293441, ENST00000382100, ENST00000373552, ENST00000256460, ENST00000382099, ENST00000424173, ENST00000412050, ENST00000428762, ENST00000504921, ENST00000537886, ENST00000348639, ENST00000244458, ENST00000525166, ENST00000323927, ENST00000404537, ENST00000532820, ENST00000389645, ENST00000411582, ENST00000233638, ENST00000274289, ENST00000543707, ENST00000245304, ENST00000367123, ENST00000281821, ENST00000455551, ENST00000555765, ENST00000298910, ENST00000378214, ENST00000550008, ENST00000555818, ENST00000298892, ENST00000430948, ENST00000447531, ENST00000408936, ENST00000263238, ENST00000252891, ENST00000316626, ENST00000549414, ENST00000241069, ENST00000372462, ENST00000425244, ENST00000347547, ENST00000359629, ENST00000306052, ENST00000395540, ENST00000395542, ENST00000395863, ENST00000395805, ENST00000614585, ENST00000347193, ENST00000395747, ENST00000395749, ENST00000359082, ENST00000417045, ENST00000356444, ENST00000409079, ENST00000335712, ENST00000556210,	4.12	8.50 E-7	7. 40 E-3

										ENST00000603926, ENST00000556161, ENST00000452389, ENST00000368476, ENST00000368407, ENST00000368406, ENST00000585482, ENST00000359265, ENST00000412389, ENST00000331758, ENST00000317338, ENST00000226730, ENST00000368983, ENST00000372735, ENST00000258682, ENST00000409938, ENST00000438547, ENST00000339554, ENST00000409822, ENST00000619168, ENST00000356842, ENST00000339861, ENST00000448867, ENST00000304613, ENST00000440254, ENST00000515385, ENST00000338448, ENST00000341223, ENST00000436636, ENST00000341394, ENST00000380180, ENST00000313401, ENST00000317968, ENST00000510942, ENST00000420101, ENST00000261726, ENST00000242462, ENST00000530820, ENST00000546411, ENST00000309909, ENST00000309989, ENST00000392521, ENST00000360648, ENST00000396751, ENST00000338963, ENST00000318007, ENST00000265136, ENST00000360264, ENST00000457475, ENST00000457714, ENST00000535468, ENST00000259371, ENST00000506154, ENST00000391813, ENST00000346990, ENST00000535378, ENST00000371941, ENST00000371953, ENST00000265997, ENST00000441453, ENST00000369780, ENST00000618146, ENST00000437259, ENST00000265663, ENST00000371553, ENST00000340208, ENST00000420987, ENST00000437473, ENST00000265348, ENST00000265334, ENST00000371475, ENST00000371474, ENST00000371473, ENST00000371472, ENST00000371454,			

										ENST00000457056, ENST00000379989, ENST00000379996, ENST00000514028, ENST00000514015, ENST00000218224, ENST00000538621, ENST00000381232, ENST00000292535, ENST00000620693, ENST00000435532, ENST00000255030, ENST00000508216, ENST00000620245, ENST00000508342, ENST00000381569, ENST00000260386, ENST00000559658, ENST00000326828, ENST00000353625, ENST00000503974, ENST00000419336, ENST00000374431, ENST00000248975, ENST00000454014, ENST00000358550, ENST00000358510, ENST00000358707, ENST00000361589, ENST00000319283, ENST00000302913, ENST00000485972, ENST00000450295, ENST00000361229, ENST00000358741, ENST00000354412, ENST00000302190, ENST00000264431, ENST00000431601, ENST00000514743, ENST00000370830, ENST00000264601, ENST00000264051, ENST00000378993, ENST00000378845, ENST00000297518			
