# Supplementary material for: Neurodevelopment Genes Encoding Olduvai Domains Link Myalgic Encephalomyelitis to Neuropsychiatric Disorders
Source: Diagnostics (Basel). 2025 Jun 17;15(12):1542. doi: 10.3390/diagnostics15121542 (PMC12191824; doi:10.3390/diagnostics15121542)
Supplement: Supplementary file 1 [file diagnostics-15-01542-s001.zip › Supplement.Figs.pdf]

Supplementary Figures (Arcos-Burgos *et al*. - ME/CFS and Olduvai Domains).

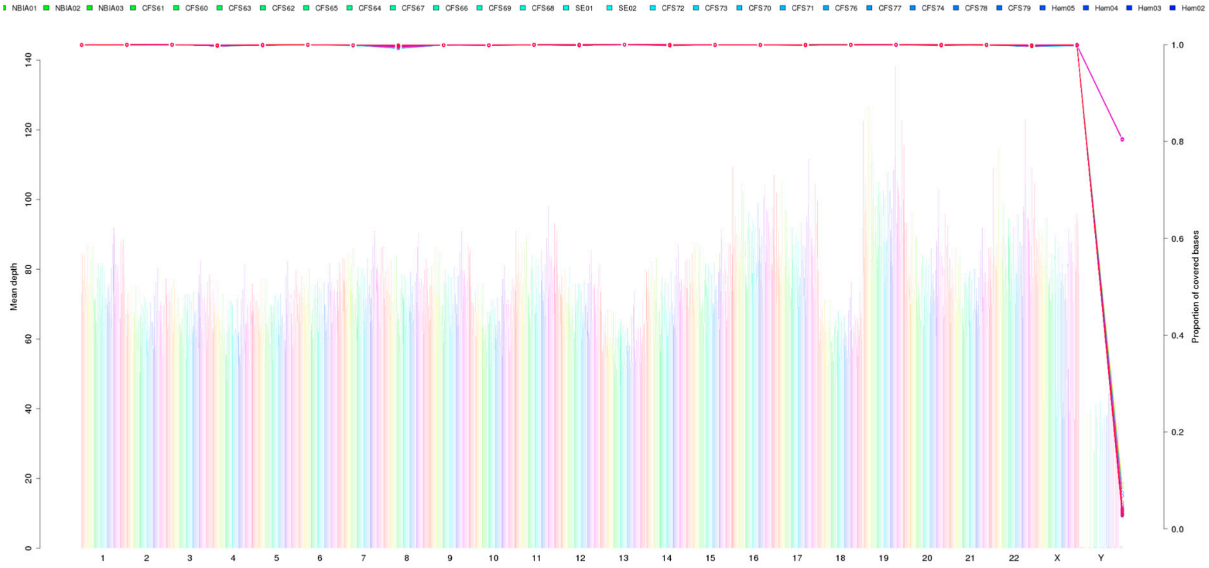

Figure S1. Average sequencing depth (bar plot) and coverage (dot-line plot) in each chromosome investigated (exome capture) from Australian ME/CFS patients (n = 77) assessed and confirmed via the International Consensus Criteria (ICC).

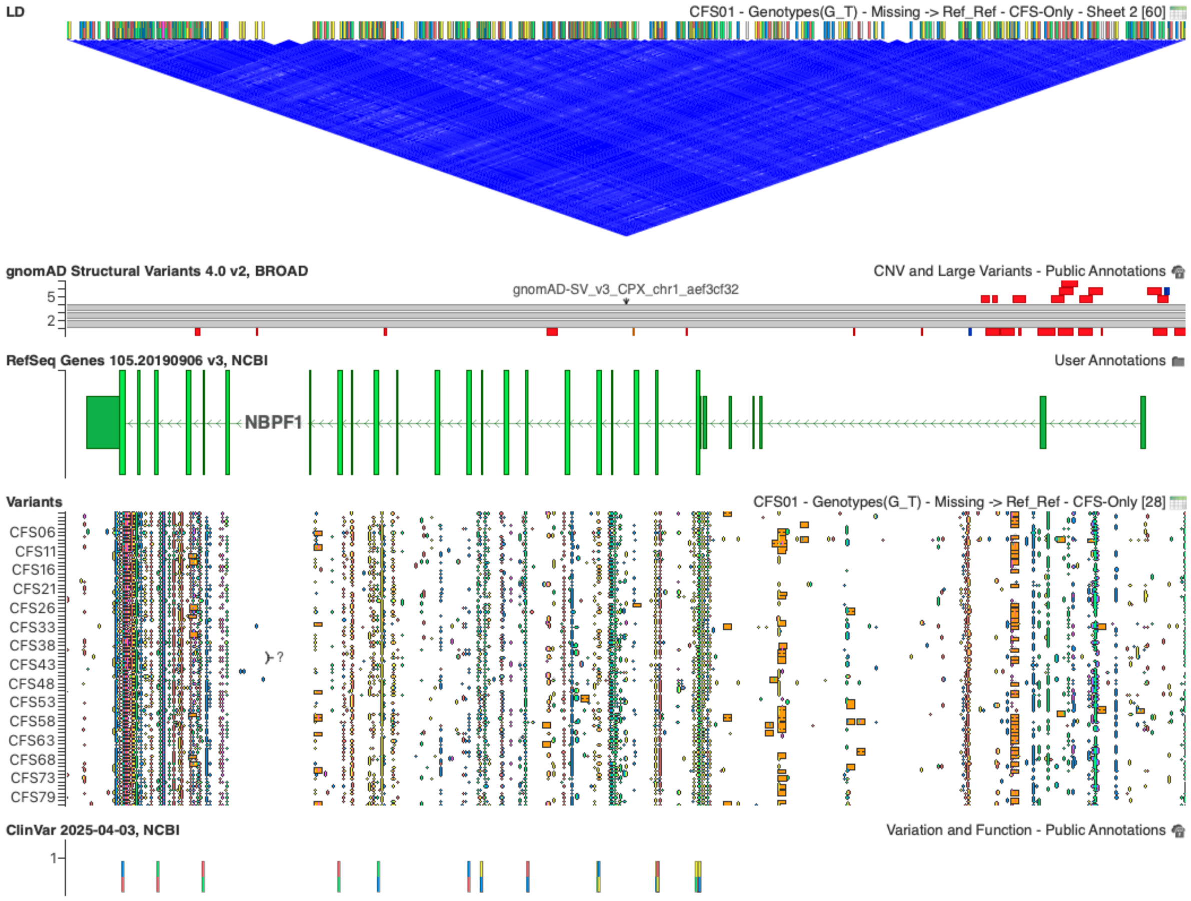

Figure S2. High resolution of SNPs and CNVs spanning the NBPF1 gene from the Australian ME/CFS patient cohort investigated post exome capture.

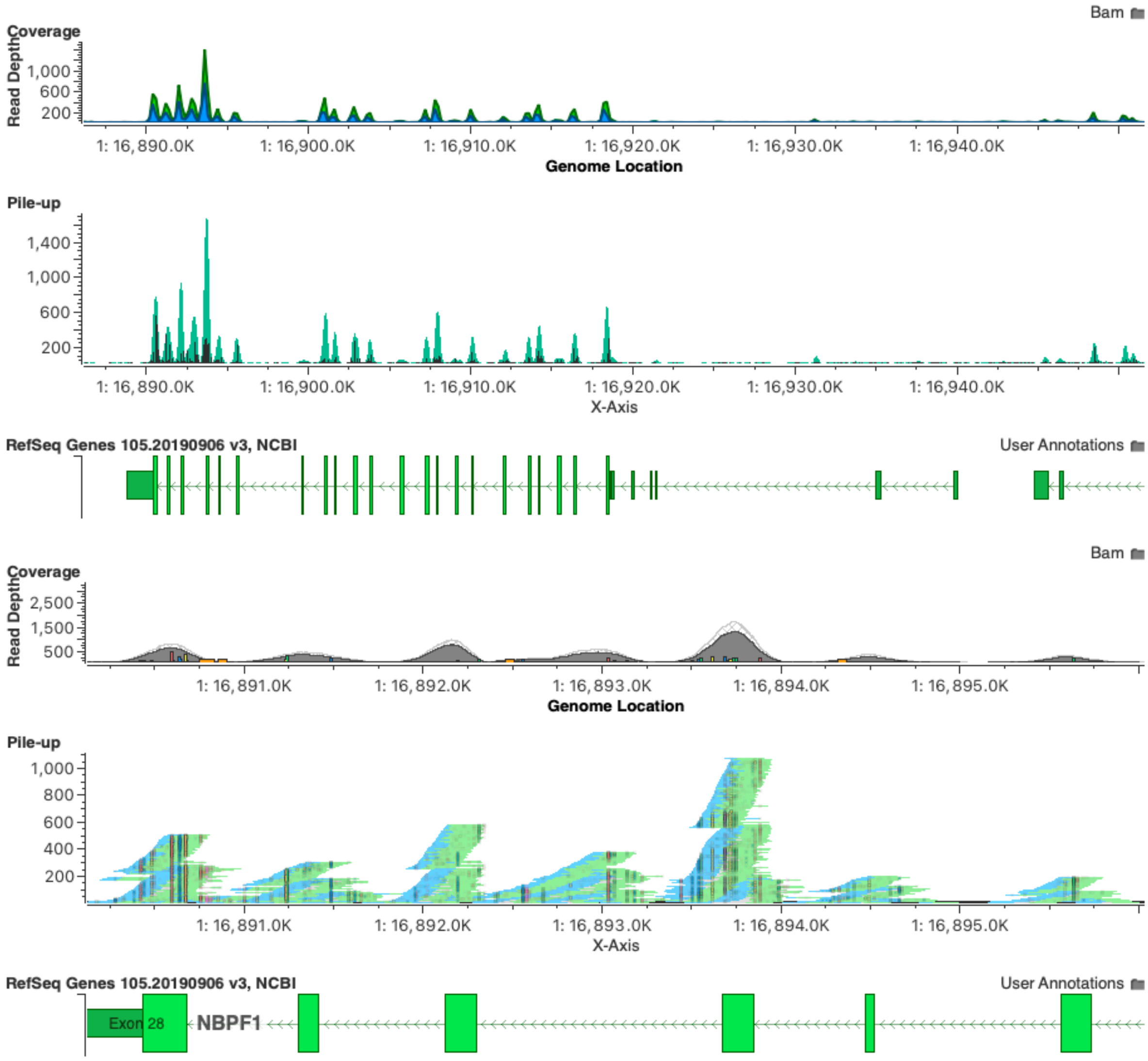

Figure S3. Coverage, depth and pile up of readings spanning NBPF1, with a zoom in over the last 6 exons of the gene at the 3′ region detected in the investigated Australian ME/CFS cohort.
